# Migraine with Focal Cortical Dysplasia: A Case Report

**DOI:** 10.3390/neurolint18060104

**Published:** 2026-05-26

**Authors:** Michal Fila, Janusz Blasiak

**Affiliations:** 1 Department of Developmental Neurology and Epileptology, Polish Mother’s Memorial Hospital Research Institute, 93-338 Lodz, Poland; michal.fila@iczmp.edu.pl; 2 Faculty of Medicine, Collegium Medicum, Mazovian Academy in Plock, 09-420 Plock, Poland

**Keywords:** migraine, focal cortical dysplasia, left occipital lobe, case report

## Abstract

**Background/Objectives**: Migraine may be associated with structural changes in the brain, including the cerebellum and brainstem. Some of these changes reflect the brain’s plasticity in adapting to migraine-related alterations, but others may influence the severity of migraines and resistance to treatment. Some studies report changes in cortical thickness among migraine patients, and focal cortical dysplasia (FCD) has been considered a possible cause of these changes. We argued that FCD could contribute to the development of migraine and the severity of its symptoms. To date, there has been no consistent report of FCD occurring in migraine patients. **Case**: A 29-year-old woman presented with a history of at least 19 years of high-frequency episodic migraine without aura. She experienced motion sickness during childhood and adolescence. Her condition worsened last year, evolving into chronic migraine, which was partially controlled by medications such as amitriptyline and rizatriptan, leading to high-frequency episodic migraines. An MRI conducted in 2024 showed a small area of signal abnormality in the left occipital lobe, believed to represent cortical dysplasia. A follow-up MRI after three months showed no changes in this area. She is currently diagnosed with high-frequency episodic migraine and demonstrated severe migraine-related disability, with a MIDAS score of 25, and a severe impact on daily functioning, with a HIT-6 score of 65. **Conclusions**: The case involves a worsening migraine that was somewhat alleviated by a pharmacological intervention. FCD may contribute to brain hyperexcitability in this case and her motion-related problems during childhood and adolescence. FCD could also play a role in the increasing severity of her migraines and her partial resistance to medication.

## 1. Introduction

Migraines display a wide range of symptoms, from mild discomfort to severe disabilities, such as those linked to chronic migraine with aura, which restricts many professional and daily activities. However, the mechanism behind this significant variation within a single disease remains poorly understood. “The migraine generator” is a concept that refers to specific brain regions, including the periaqueductal gray (PAG), dorsolateral pons, and rostral brainstem, forming the central hub for migraine development [1]. A modification of the migraine generator concept suggests that the brainstem oscillates between a state in which incoming cerebrovascular inputs can trigger head pain and a state in which those same inputs are blocked from reaching the cortex, thereby preventing head pain [2]. This concept further evolved, shifting the focus from a single generator to a “high-risk area” in the brain that arises from the interaction of various physiological and psychological factors, including unstable or depressive mood, restless sleep, and autonomic imbalance (reviewed in [3]). However, it is not known what sustains the activity of the migraine generator to trigger recurring migraine attacks over the years.

The concept of “migraine generator” and its modifications suggest viewing migraine as a benign condition. While migraine may be linked with structural changes in the brain, our understanding of the functional significance of this association, especially regarding potential links between such changes and symptom severity, remains limited. This is primarily because only a small fraction of migraine patients undergo MRI examinations. To interpret changes in cortical thickness in migraine patients, focal cortical dysplasia (FCD) was considered as a potential cause for increased cortical excitability [4]. A series of cases with migraine-like visual aura linked to focal cerebral lesions were presented [5]. Similarly, a few cases of migraine-like positive visual phenomena linked to focal cortical lesions with undetectable field defects were described [6].

Focal cortical dysplasia is causally linked to some drug-resistant epilepsies and is often the reason for epilepsy surgery [7]. A recent meta-analysis showed an 80% increase in the lifetime prevalence of epilepsy among patients with migraine compared to those without migraine [8]. Therefore, FCD might be a component of structural changes observed in migraine patients, especially those with worsening migraines, and could contribute to the migraine-epilepsy connection. We have recently provided additional arguments regarding the potential role of FCD in migraine development; however, to date, there has been no consistent evidence of FCD in migraine [9].

## 2. Case Presentation

The 29-year-old cisgender woman has been experiencing migraines since she was 10 years old or younger. She reports no history of epilepsy or seizure-like attacks. She suffered from motion sickness between the ages of 9 and 12. She is sensitive to light, including sunlight, which often triggers migraines. She has never smoked and has no history of hypertension or other cardiovascular conditions. She used hormonal contraception from ages 18 to 20, but it seemed to worsen her migraines, making them more frequent and severe, leading her to stop. Lifestyle changes appeared to help reduce migraine. In early 2024, her headache frequency increased, occurring nearly every day, about 5–6 days per week. In June 2024, she began taking medications: amitriptyline (10 mg) for prevention and rizatriptan (10–20 mg daily, no more than 5 days per month) to abort attacks. Despite this, many headache episodes continue. She has never used anti-CGRP therapy, nor has she been offered or suggested to undergo such treatment.

Once she started taking the medications, her migraines decreased from over 20 days per month to 5–10 days per month. This improvement has continued over the last year. Due to worsening migraines, she was ordered to have an MRI. Her migraine attacks became more frequent and severe over the past few years, and as a result, she was scheduled for an MRI, which was performed twice in 2024.

The MRI scans from the initial examination show FCD in the left occipital lobe, marked with arrows on the T2-weighted Fluid-Attenuated Inversion Recovery (T2-FLAIR) sequence (Figure 1A,B). There may be a band of hyperintense signal extending from the cortex into the white matter, known as the “transmantle sign.” A small confluent area of T2-FLAIR hyperintensity is present in this location, with blurring of the gray-white junction. Figure 1C,D show blurring of the cortical-subcortical boundary on T1-weighted MRI, but FCD appears more distinct in T2-FLAIR. In the second MRI examination, performed 3 months after the first, no changes were observed in the small subcortical T2-FLAIR hyperintensity in the left occipital lobe, which is presumed to represent FCD.

Currently, she has a MIDAS score of 25, corresponding to Grade IV (severe migraine-related disability), and a HIT-6 score of 65, indicating a severe impact of migraine on daily functioning. In summary, the patient has a case of worsening migraine, which was partly alleviated by medications. A stable FCD is present in her brain.

## 3. Discussion and Conclusions

The patient presents a case of migraine with severe symptoms, serving as a serious burden, lasting for years, and significantly worsening over the last few years, only partly mitigated pharmacologically. The patient shows structural changes in the brain, suggesting FCD. As we mentioned, we have not found consistent evidence of the occurrence of FCD in migraine, but we want to emphasize once more that only a small fraction of migraine patients undergo an MRI examination. Definitive diagnosis of FCD requires histopathological examination of resected brain tissue. In patients who do not undergo epilepsy surgery, the diagnosis remains presumptive, based on clinical, electroencephalographic, and neuroimaging findings. MRI is the principal non-invasive tool for identifying FCD, but it cannot reliably substitute for histopathological confirmation.

The localization of the suspected focal cortical dysplasia (FCD) to the occipital lobe is of potential interest in the context of migraine pathophysiology. The occipital cortex, particularly the visual cortex, plays a central role in migraine, especially in migraine with aura, where CSD is thought to originate and propagate across this region. Furthermore, neuroimaging studies have consistently demonstrated altered excitability and functional connectivity within the visual network in migraine patients, even in the absence of aura. However, current evidence does not support a consistent lateralization of these changes to either the left or right hemisphere. Occipital involvement in migraine is typically bilateral and dynamic, with substantial inter- and intra-individual variability. Therefore, while the presence of an occipital cortical abnormality in this patient may be neurobiologically relevant, any direct association between the left-sided localization of the lesion and migraine pathogenesis remains speculative.

The immediate question about the patient’s case is whether FCD might be a cause or a consequence of migraine. This question addresses the fundamental features of migraine pathophysiology, in which two aspects are interconnected: a migraine-susceptible brain and migraine triggers that only affect patients with such brains [10]. Migraine-inducing substances, such as vasoactive peptides including calcitonin gene-related peptide (CGRP) and pituitary adenylate cyclase-activating peptide (PACAP), infused intravenously, trigger migraine attacks only in individuals with migraines. In contrast, non-migraine subjects either are not affected or experience mild symptoms. As mentioned, increased cortical thickness has been observed in migraine patients with and without aura [11,12]. Such an increase in the cortex may be highlighted by a local rise in the number of neurons and/or glial cells. These local dysplasias can cause the cortex to become hyperexcitable, which supports the hypothetical and evidence-based hyperexcitability observed in the brains of migraine patients [13,14].

However, it is debated whether the site of action of neuroactive peptides is outside or inside the central nervous system [10]. If the latter is true, these substances must enter the brain, crossing all barriers between the brain and the periphery. In this case, substances from the periphery might encounter brain changes that facilitate their transport in individuals susceptible to migraines. Such changes could be functional, structural, or both. Therefore, FCD could be a factor that helps vasodilatory substances enter the brain. On the other hand, migraine is a condition with a wide range of symptoms—from rare, mild headaches that do not interfere with daily life to chronic migraines with aura, which can cause long periods of serious disability and prevent many professional and lifestyle activities. The question remains: what causes this variation in migraine symptoms? Naturally, the strength of triggers might be a short-term cause of such differences. However, this explanation does not apply to cases of migraine with a heavy burden, as triggers are usually identified and avoided by those affected. Therefore, when considering a migraine-susceptible brain in relation to migraine severity, we should probably think of it as a spectrum of more or less susceptible brains.

It should be emphasized that conventional structural MRI sequences (T1-, T2-, and FLAIR-weighted imaging), as in the present study, are not designed to visualize the hallmark pathophysiological mechanisms of migraine. Migraine is primarily a functional brain disorder involving dynamic processes such as CSD, trigeminovascular activation, and altered network connectivity, which are not directly detectable on routine structural imaging. Instead, MRI in this clinical setting is principally used to exclude secondary causes of headache rather than to confirm migraine or assess its severity. Advanced neuroimaging techniques, such as functional MRI or perfusion imaging, have demonstrated transient changes in cortical activity and cerebral blood flow during migraine attacks, particularly in association with aura, as well as altered functional connectivity in pain- and sensory-processing networks. However, these findings remain indirect, variable across patients, and are not currently applicable to routine clinical assessment or to individual-level correlation with disease severity. In the present case, the purpose of including MRI data was therefore not to characterize migraine-related brain changes, but to document the presence of a focal cortical abnormality suggestive of focal cortical dysplasia (FCD). While MRI can raise suspicion for FCD, particularly in the presence of characteristic structural features, it also does not provide definitive histopathological confirmation. Consequently, no direct inference can be made from the available MRI data regarding the underlying migraine pathophysiology or its severity.

Emerging evidence suggests that hyperexcitability or abnormal development in motion-sensitive regions of the visual cortex—especially the middle temporal visual area, V5/MT—may contribute to motion sickness in children, although the connection remains unconfirmed [15]. Our patient experienced motion sickness in childhood, and we cannot exclude a connection between these symptoms and FCD. Although the patient did not experience visual aura or epileptic seizures, she did exhibit light sensitivity and a history of motion sickness. These features may be associated with abnormal development in motion-sensitive regions of the visual cortex. It is also worth investigating whether anti-CGRP treatment could reduce migraine symptoms more effectively than amitriptyline and rizatriptan.

In conclusion, the case involved long-lasting worsening migraines associated with some motion disturbances, and pharmacological treatment resulted in only moderate improvement. The focal cortical dysplasia identified in the case may contribute to the brain hyperexcitability and the motion-related problems she experienced during childhood.

## Figures and Tables

**Figure 1:**
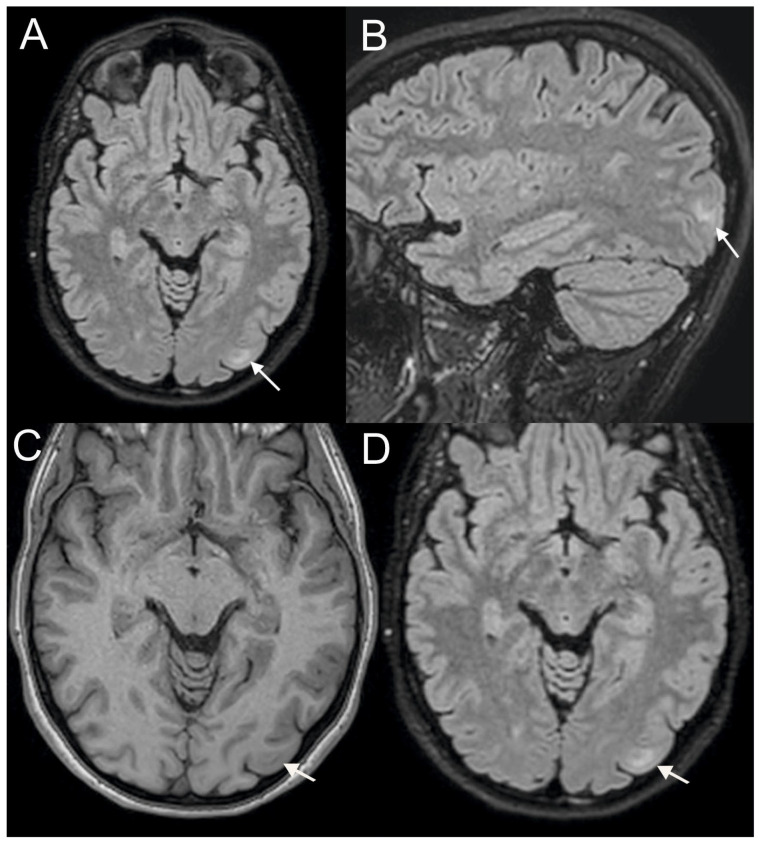
Magnetic resonance images of the brain showing different views and sections. (**A**) Axial FLAIR image showing hyperintensity in the left temporal lobe (arrow). (**B**) Sagittal FLAIR image showing hyperintensity in the left parietal lobe (arrow). (**C**) Axial T1-weighted image showing hypointensity in the left temporal lobe (arrow). (**D**) Axial FLAIR image showing hyperintensity in the right occipital lobe (arrow).

## Data Availability

The data presented in this study are available on request from the corresponding author. The data are not publicly available due to privacy and ethical restrictions.

## Abbreviations

CGRP	Calcitonin gene-related peptide
FCD	Focal cortical dysplasia
HIT-6	Headache Impact Test-6
MIDAS	Migraine Disability Assessment Scale
MRI	Magnetic Resonance Imaging
PACAP	Pituitary adenylate cyclase-activating peptide
T2-FLAIR	T2-weighted Fluid-Attenuated Inversion Recovery
